# *De Novo* Variants in the *DYNC1H1* Gene Associated With Infantile Spasms

**DOI:** 10.3389/fneur.2021.733178

**Published:** 2021-11-05

**Authors:** Haipo Yang, Pan Gong, Xianru Jiao, Yue Niu, Qiujun Zhou, Yuehua Zhang, Zhixian Yang

**Affiliations:** Department of Pediatrics, Peking University First Hospital, Beijing, China

**Keywords:** infantile spasms, epilepsy, malformations of cortical development, *DYNC1H1* gene, intellectual disability

## Abstract

**Objective:** The *DYNC1H1* gene is related to a variety of diseases, including spinal muscular atrophy with lower extremity–predominant 1, Charcot–Marie–Tooth disease type 2O, and mental retardation, autosomal dominant13 (MRD13). Some patients with *DYNC1H1* variant also had epilepsy. This study aimed to detect *DYNC1H1* variants in Chinese patients with infantile spasms (ISs).

**Methods:** We reviewed clinical information, video electroencephalogram (V-EEG), and neuroimaging of a newly identified cohort of five patients with *de novo DYNC1H1*gene variants.

**Results:** Five patients with four *DYNC1H1*variants from four families were included. All patients had epileptic spasms (ESs), the median age at seizure onset was 7.5 months (range from 5 months to 2 years 7 months), and the interictal V-EEG results were hypsarrhythmia. Four of five patients had brain magnetic resonance imaging (MRI) abnormalities. Four *de novo DYNC1H1* variants were identified, including two novel variants (p.N1117K, p.M3405L) and two reported variants (p.R1962C, p.F1093S). As for the variant site, two variants are located in the tail domain, one variant is located in the motor domain, and one variant is located in the stalk domain. All patients had tried more than five kinds of antiepileptic drugs. One patient has been controlled well by vigabatrin (VGB) for 4 years, and another patient by VGB and steroids for 1.5 years. The other three patients still had frequent ESs. All patients had severe intellectual disability and development delays.

**Significance:** IS was one of the phenotypes of *DYNC1H1* variants. Most patients had non-specific brain MRI abnormality. Two of four *DYNC1H1* variants were novel, expanding the variant spectrum. The IS phenotype was related to the variant's domains of *DYNC1H1* variant sites. All patients were drug-refractory and showed development delays.

## Introduction

**Figure 1:**
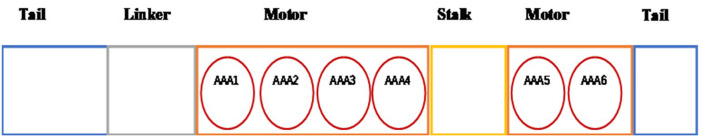
The protein regions of DYNC1H1.

The *DYNC1H1* gene located in 14q32.31 encodes for dynein cytoplasmic one heavy chain 1. It is a large protein of 530 kDa and 4,646 amino acids (aa), which is highly conserved and has a few housekeeping roles (1). *DYNC1H1*comprises four major protein regions (Figure 1), that is, tail domains (aa residues 1–1,373 and 4,222–4,646), linker domain (aa 1,374–1,867), motor domains with AAA domains (ATPases associated with a variety of cellular activities, aa 1,868–3,168 and 3,553–4,221), and the stalk or microtubule-binding domain (MTBD, aa 3,169–3,552) (2).

*DYNC1H1* is highly intolerant to missense change (3). Heterozygous variants in the *DYNC1H1* gene have been associated with a variety of diseases. In 2010, Harms et al. first described dominant spinal muscular atrophy (SMA) with lower extremity with *DYNC1H1* variant (4). Weedon et al. identified a *DYNC1H1* variant in a large pedigree with autosomal dominant axonal Charcot–Marie–Tooth disease in 2011 (5). Willemsen et al. and Poirier et al. reported *DYNC1H1* variants caused severe intellectual disability with neuronal migration defects and malformations of cortical development (MCDs) (6, 7). Different *DYNC1H1* gene variant sites were related to different phenotypes. However, patients with the same variant might also have different phenotypes; for example, p.Arg598Cys variant was found in one patient diagnosed as having SMA, and the other patient diagnosed with myopathy (8, 9).

Some patients with *DYNC1H1* variants manifest epilepsy. In 2013, Poirier et al. reported eight patients with *DYNC1H1* gene variant when studying the mutated genes of patients with MCD and microcephaly, among whom seven patients developed epileptic seizures, and one was diagnosed with Lennox-Gastaut syndrome (LGS) syndrome (7). In 2020, Amabile et al. summarized 103 *DYNC1H1* variants in 200 patients with neurological developmental phenotypes across 143 unique families (10). Seizures were found in 18.5% (37/200) of patients. Here, we report five infantile spasm (IS) patients with *DYNC1H1*variants and characterize in detail the clinical phenotype, brain magnetic resonance imaging (MRI) features, and response to treatment and outcome.

## Methods

### Patients

Five patients in four families with *DYNC1H1* variants were retrospectively recruited from the Department of Pediatrics, Peking University First Hospital, from June 2017 to October 2020. This study was approved by the Peking University First Hospital Medical Ethics Committee. Information about the age at epileptic spasm (ES) onset, developmental milestones, neurological status, family history, video electroencephalogram (V-EEG), brain MRI results, treatment, and outcomes was collected in the clinic. Brain MRI and V-EEG were reviewed by a neuroradiologist and neurophysiologist, respectively. Patients were followed up at the pediatric neurology clinic or by telephone.

DNAs (3 μg) extracted from peripheral blood from probands and their parents were analyzed using whole-exome sequencing. Variants were checked with population databases gnomAD (http://gnomad.broadinstitute.org/) and evaluated using Polyphen2, SIFT, and Variant Taster. Variant pathogenicity was interpreted according to the American College of Medical Genetics (ACMG) guidelines (11). The variants were further confirmed by Sanger sequencing.

### Literature Review

A systematic research of articles published in PubMed registered from 2012 to 2021, was performed. All the researched articles are based on the following terms “DYNC1H1.” The relevance of each result was determined, and references were reviewed to identify missing studies. All the epilepsy phenotype, epilepsy onset age, treatment, and prognosis are summarized in Supplementary Table 1.

## Results

### Clinical Features

Clinical features of affected individuals with *DYNC1H1* variants are summarized in Table 1.

**Table 1:** The clinical information and gene variants of five patients.

	**Patient 1**	**Patient 2**	**Patient 3**	**Patient 4**	**Patient 5**
Sex	Female	Female	Female	Female	Female
Current age	5 y	2 y 9 m	3 y	3 y	3 y
Gestation	At term	At term	At term	At term	At term
Birth history	Normal	Normal	ABO hemolytic disease of newborn	ABO hemolytic disease of newborn	Normal
Age at seizure onset	10 m	5 m	7.5 m	7.5 m	2 y 7 m
Seizure type	Spasms	Spasms	Spasms	Spasms	Spasms
AEDs	ACTH, VPA, ketogenic diet, ZNS, TPM, LEV	LEV, TPM, ACTH, VGB, steroids	LEV, TPM, VPA, CLB, VGB, LTG	LEV, TPM, VPA, CLB, VGB, LTG	VPA, TPM, CAP, LTG, VGB, steroids, ketogenic diet
Treatment	VGB controlled 4 y	VGB+ steroids controlled 1.5 y	Uncontrolled	Uncontrolled	Uncontrolled
EEG	Hypsarrhythmia	Hypsarrhythmia	Hypsarrhythmia	Hypsarrhythmia	Hypsarrhythmia
Brain MRI	White matter dysplasia	Enlarged bilateral frontal gyrus, thickened cortex, and reduced subcortical white matter	Enlarged bilateral hemispheric gyrus, ventricles, and corpus callosum dysplasia	Enlarged bilateral hemispheric gyrus, ventricles, and corpus callosum dysplasia	Normal
Genetic test	DYNC1H1, *de novo*, c.3351C>G (p.N1117K) (novel)	DYNC1H1 *de novo*, c.5884C>T (p.R1962C) (reported)	DYNC1H1 *de novo*, c.10213A>C (p.M3405L) (novel)	DYNC1H1 *de novo*, c.10213A>C (p.M3405L) (novel)	DYNC1H1 *de novo*, c.3278T>C (p.F1093S) (reported)
Psychomotor development	Severe delay	Severe delay	Severe delay	Severe delay	Severe delay
Sitting	1 y 5 m	No	No	No	8 m
Walking	5 y	No	No	No	1 y 8 m
Speech	No	No	No	No	No

### Seizures, EEG, and Brain MRI Information

All five patients were from full-term birth. Patients 3 and 4 were dizygotic twins. Patient 5 had two febrile convulsions at 1½ and 2 years old. All patients had ES. For patients 1–4, the minimum seizure onset age was 5 months, and the maximum onset age was 7.5 months. As for patient 5, the first seizures occurred at the age of 2 years 7 months. All patients have tried at least five kinds of antiepileptic drugs (AEDs), including adrenocorticotropic hormone (ACTH), valproate (VPA), zonisamide (ZNS), topiramate (TPM), levetiracetam (LEV), vigabatrin (VGB), steroids, and a ketogenic diet. The ES of patient 1 was controlled by VGB at the age of 1.5 years for more than 4 years, albeit multifocal discharges on EEG were still observed until the last follow-up. For patient 2, ES was controlled by VGB and steroids at the age of 1 year 5 months for 1.5 years. The ES was not controlled for patients 3, 4, and 5.

**Figure 2:**
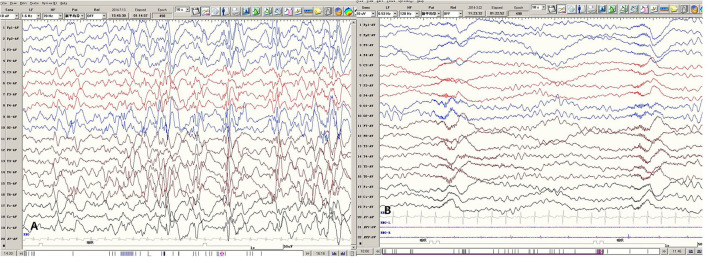
Representative EEGs of Patient 1. **(A)** The Interictal hypsarrhythmia EEG pattern. **(B)** The ES ictal EEG pattern.

**Figure 3:**
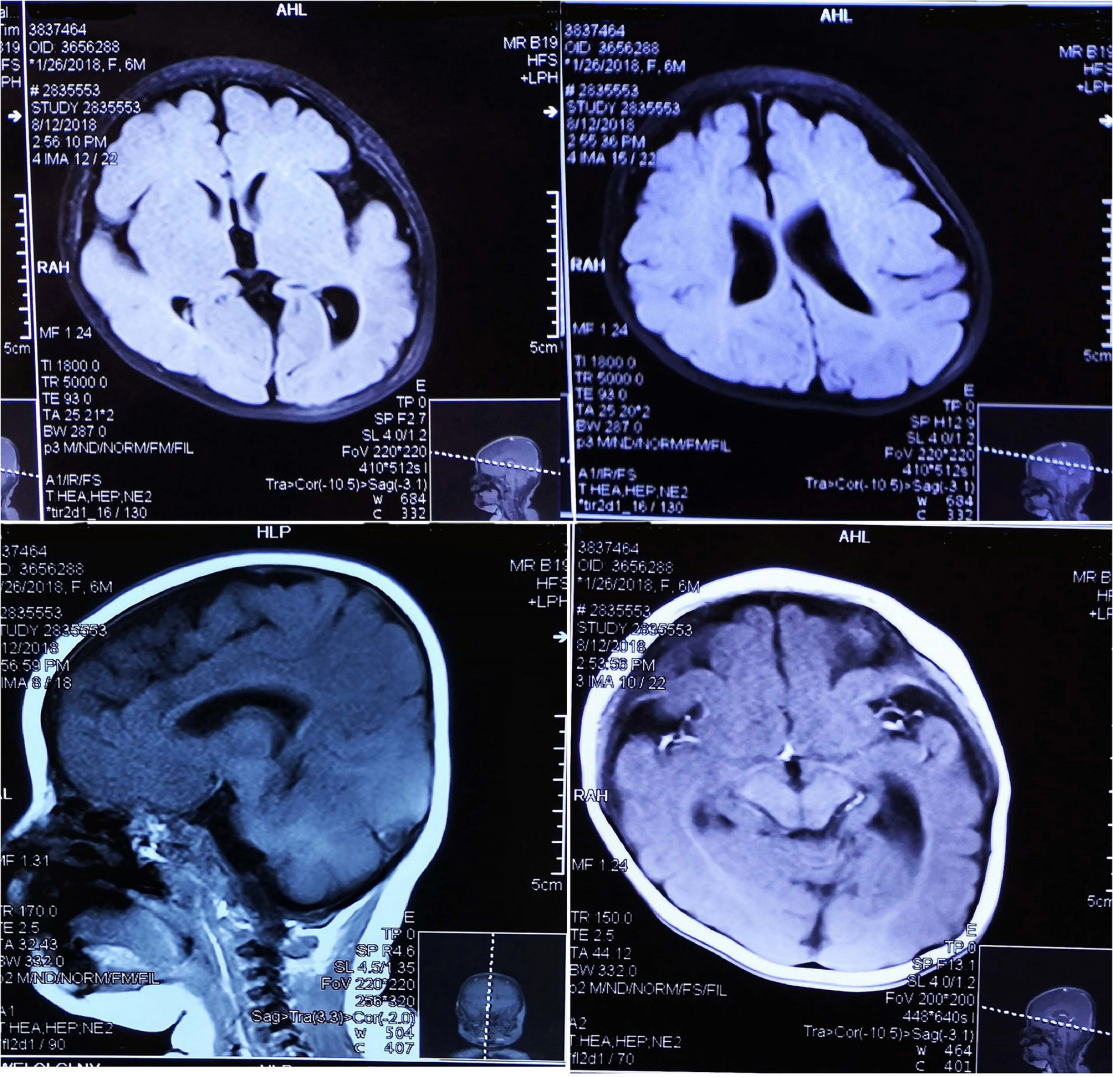
The brain MRI of patient 3 showed MCD.

EEG data for five patients all showed hypsarrhythmia (Figure 2). Brain MRI was abnormal in patients 1–4 and normal in patient 5. Brain MRI showed white matter dysplasia and developmental regression in patient 1; enlarged bilateral frontal gyrus, thickened cortex, and reduced subcortical white matter in patient 2; and enlarged bilateral hemispheric gyrus, ventricles, and corpus callosum dysplasia in patients 3 and 4 (Figure 3).

### Genetic Analysis

Four *de novo* variants were identified in five patients from four families. Two variants (p.N1117K, p.M3405L) were novel, and two (p.R1962C, p.F1093S) were previously reported (12, 13). All four variants located in different domains, including p.N1117K in the distal dimerization domain of the protein (tail domains), p.R1962C in theAAA1 of the protein (motor domain), p.M3405L in the AAA4 of the protein (stalk or MTBD, motor domain), and p.F1093S in the AAA1 of the protein (tail domains). All the detected variants were pathogenic according to the ACMG criteria.

### Neurodevelopment

All patients had severe developmental delays and intellectual disabilities, but no developmental scales were available. Among them, only two patients could walk independently at the last follow-up. The motor development of patients 3 and 4 underwent a retrograde process. Patients 3 and 4 could control their necks at the age of 5 months. After seizure onset, they could not hold their head well and could not sit until 3 years old. At the last follow-up, all patients still had language backward. Only patient 5 could understand simple instructions and speak a few words, but he had from significant language regression to unable to speak after ES onset. The other four patients could not speak any words.

### Results on Literature Search

A total of 18 articles found *DYNC1H*1 gene associated with epilepsy. Forty-three patients were reported to have epilepsy. In these 43 patients, the epilepsy phenotype was described in 44.2% (19/43), including focal seizure, myoclonic seizures, tonic seizures, atonic seizures, generalized tonic–clonic seizures, and IS. EEG results were reported in 11.6% of patients (5/43), including multifocal epileptiform discharges; interictal EEG showed waxing and waning of waves in the frontal, temporal, and occipital areas, and high-amplitude rhythmic waves were frequently observed; generalized spike-and-wave complexes and irregular polyspikes and slow waves, predominantly in the left frontal area; an attenuated background of mixed theta and delta frequencies; and semiperiodic spike and slow wave activity in the right temporal occipital region and generalized slowing. Treatment was described in 48.8% of patients (21/43). Nine patients were seizure-free or had controlled epilepsy. Ten patients had uncontrolled epilepsy or were therapy-refractory. The other three patients did not have their therapy result.

## Discussion

We reported five patients with IS from four families with *de novo DYNC1H1* variants, including two novel and two previously reported variants. We also reviewed previous literature about *DYNC1H1* gene–associated epilepsy.

In 2018, Palmer et al. reported one patient with p.R1962C variant had IS (12). His EEG revealed an abnormal background and multifocal epileptiform activity. His brain MRI showed pachygyria (12). This patient had severe developmental delay and intellectual disability, and he was diagnosed with an autism spectrum disorder (12). Patient 2, carrying the same p.R1962C variant, manifested ES, MCD, severe developmental delay, and intellectual disability, similar to the reported patient (12). However, Poirier et al. reported a 19-year-old patient with p.R1962C variant who had a focal seizure, severe developmental delay, intellectual disability, and the predominant postpachygyria (7). Therefore, patients with the same *DYNC1H1* variant might have different seizure types. In 2016, Helbig et al. reported a patient diagnosed as having IS with the variant site p.F1093S. The same variant was found in patient 5 (13). Besides IS, several epileptic phenotypes have been reported in patients with *DYNC1H1* variants, such as focal onset epilepsy, myoclonic epilepsy, and atonic seizures (2, 6, 7, 12–17). All the reported epileptic phenotypes are summarized in Supplementary Table 1.

Patients with pachygyria carrying *DYNC1H1* variants manifested epilepsy (10, 14). Abnormal brain MRI was observed in our four patients, including three MCD and one white matter dysplasia. *DYNC1H1* is known to bind both bicd cargo adaptor (BICD2), a microtubule motor adaptor associated with SMA lower extremity–predominant 2, and lissencephaly 1 (LIS1), a dynein regulator associated with MCD/lissencephaly (18), which might explain why the variant of *DYNC1H1* could lead to pachygyria or MCD. For those patients with MCD, posterior predominant lesions were most common (7). In 2020, Amabile et al. found 37 patients had epileptic seizures, among whom 28 patients showed MCD (10). For our patients, MCD was mainly observed at the frontal lobe in one patient and bilateral hemispheres in two, which were different from the previous reports (7). Epileptogenic mechanisms linked to pathogenic variants of *DYNC1H1* gene were not clear; MCD may be the reason; however, patients from previous and our studies without MCD could also have epilepsy (16). In 2017, Lin et al. reported an epileptic encephalopathy patient who had *DYNC1H1* mutation through analysis of the interaction network of DYNC1H1, which showed that DYNC1H1 interacts with many epilepsy genes, such as *TBC1D24, ALDH7A1, MECP2, DEPDC5, SGCE, GRIN2B, ATP1A2, MYO5A, NBEA, CLCN4, IFT172, UBE3A, PCDH19, KCNQ3*, and so on. Thus, DYNC1H1 may cause epilepsy by affecting other epilepsy-related gene function, such as interaction with other mutations present in their genomes or environmental factor. In addition to the above speculation, the mutation of *DYNC1H1* gene itself may also cause epilepsy, because DYNC1H1 is highly conserved and takes part in a variety of intracellular functions (17).

Several studies have reported the relationship between domain location of the variants and clinical phenotype (2, 10). A previous study reported that *DYNC1H1* variants in eight patients with MCD were located in the stalk domain, AAA1, the linker region, and the tail domain (7). The study also reported that four unrelated patients with MCD disorder had *de novo* variants in the stalk domain who exhibited obvious clinical symptoms of early-onset epilepsy encephalopathy (7). Amabile et al. summarized 103 *DYNC1H1* variants and concluded that among 26 neuromuscular patients with obvious central nervous system involvement (intellectual disability, MCD, or other brain MRI abnormalities), 23 had variants located in the stem or neck domains, and only three had variants located in the motor domain (10). In contrast, of the 59 patients classified as having a primarily intellectual disability, MCD, and autism, 18 had variants located within the stem domain, five in the neck/linker, and 36 in the motor domain (10). Beecroft et al. summarized that a majority of patients with MCD had variants in the stalk of the motor domain (9). Some studies reported that variants associated with central nervous system manifestations such as intellectual disability and MCD had clustered in the motor domains (4, 18). Variants from the patients with seizures were mostly reported in the motor domain, whereas variants from the patients with behavioral abnormalities were largely reported in the beginning tail, linker, and motor domains (2). Variants from the patients with MRI abnormalities, specifically, pachygyria, were largely reported in the motor domain (2). In our study, the patient numbers were too small to conclude the relationship between the variant domain and the clinical phenotype. The *de novo* p.N1117K and p.F1093S variant of *DYNC1H1* identified in patients 1 and 5 were located in the tail domain and the stem domain of the protein. Both patients had obvious intellectual disability and development delay and severe epilepsy. The brain MRI revealed white matter dysplasia in patient 1, whereas it was normal in patient 5. As indicated earlier, the variants in patients with epilepsy were usually located in the motor domain of the protein; thus, these two patients were not consistent with the previous study (7, 10). The p.R1962C variant identified in patient 2 was located in the motor domain. She had ES and MCD, which was consistent with the previous reports (7, 19). The p.M3405L variant identified in patients 3 and 4 was in the stalk domain (MTBD), which belonged to the motor domains. Both patients had early-onset epilepsy and MCD, consistent with the previous report (2, 7, 19–21).

Until now, few cases have described the epilepsy treatment of patients with *DYNC1H1* variant in detail. In 2020, Becker et al. reported four patients with *DYNC1H1* variants had epilepsy and found that most patients remained seizure-free with single or combined anticonvulsive medication, whereas one patient had a therapy-refractory course (2). Di Donato et al. reported 13 patients with *DYNC1H1* variants complicated with epilepsy, among which seven cases had seizures controlled by drugs (14). Matsumoto et al. reported two patients with *DYNC1H1* variants who had refractory epilepsy. The patients were treated with more than five kinds of AEDs, but their epilepsy was not controlled successfully (16). In our study, all five patients tried at least five AEDs, and two patients were controlled by VGB and steroids.

In our study, all five patients had severe developmental delay and intellectual disability; in addition, three patients had development or language retrograde after seizure onset. In the previous studies, patients with intellectual disability were usually diagnosed with MRD13, who had the intellectual disability and cortical malformations (6). Four of our patients had abnormal brain MRI, which was consistent with the previous report (6). Some studies indicated that variants involved in central nervous system manifestations were clustered in the motor domains (4, 19). As for patient 5, the *DYNC1H1* variant was located in the tail domain, but her brain MRI was normal, which was not consistent with the previous reports (4, 19). Therefore, the mechanism of intellectual disability in patients with *DYNC1H1* variants still needs further research.

## Conclusion

Here we summarized the clinical features, treatment, and outcomes of five patients with *DYNC1H1* variants. IS was one of the phenotypes of *DYNC1H1* variants. Most patients had nonspecific brain MRI abnormalities. *DYNC1H1*-associated diseases had heterogeneity of gene variants and clinical phenotypes. All patients were drug-refractory, although some could be controlled lately. All patients had severe developmental delay and intellectual disability. For the present study, the data were too small to conclude the relationship between the clinical phenotypes and the variants' domain location; further study may be needed to draw a more accurate conclusion.

## Data Availability Statement

The datasets presented in this study can be found in online repositories. The names of the repository/repositories and accession number(s) can be found at: BankIt2488557 Seq1 MZ733295; BankIt2489627 Seq1 MZ736872; BankIt2489989 Seq1 MZ754976, and BankIt2490423 Seq1 MZ781301.

## Ethics Statement

Written informed consent was obtained from the minor(s)' legal guardian/next of kin for the publication of any potentially identifiable images or data included in this article.

## Author Contributions

HY and ZY designed the study, drafted the initial manuscript, and revised the manuscript. PG, XJ, QZ, YN, and YZ helped to collect and summarize data and revised the manuscript. All authors contributed to the article and approved the submitted version.

## Funding

This work was supported by National Natural Science Foundation of China (81771393 and 82171436), Beijing Natural Science Foundation (7202210), and Capital's Funds for Health Improvement and Research (2020-2-4077).

## Conflict of Interest

The authors declare that the research was conducted in the absence of any commercial or financial relationships that could be construed as a potential conflict of interest.

## Publisher's Note

All claims expressed in this article are solely those of the authors and do not necessarily represent those of their affiliated organizations, or those of the publisher, the editors and the reviewers. Any product that may be evaluated in this article, or claim that may be made by its manufacturer, is not guaranteed or endorsed by the publisher.
