# Supplementary material for: De Novo Variants in the DYNC1H1 Gene Associated With Infantile Spasms
Source: Front Neurol. 2021 Nov 5;12:733178. doi: 10.3389/fneur.2021.733178 (PMC8603382; doi:10.3389/fneur.2021.733178)
Supplement: Supplementary file 1 [file Table_1.DOCX]

Supplementary table 1-Epilepsy caused by DYNC1H1 gene mutation which was reported in previous literatures

|  | variants | Seizure onset age | Seizure phenotype | Diagnosed | EEG | Treatment | Reference |
| --- | --- | --- | --- | --- | --- | --- | --- |
| 1 | p.E561G | 5 y | / | / | / | / | Jamaur. 2014 |
| 2 | p.L3478F | / | Focal seizure | / | / | Seizure free | Becker, 2020 |
| 3 | p.E2294K | / | Focal seizure | / | / | therapy-refractory |  |
| 4 | p.P3173R | / | Focal seizure | / | / | Seizure free |  |
| 5 | p.E666D | / | Focal seizure | / | / | Seizure free |  |
| 6 | p.del659-662 | / | / | Early onset epilepsy | / | / | Poirier, 2013 |
| 7 | p.K129I | / | / | Late onset epilepsy | / | / |  |
| 8 | p.K3336N | / | / | Early onset epilepsy | / | / |  |
| 9 | p.R3384 | / | / | Early onset epilepsy | / | / |  |
| 10 | p.R3344Q | / | / | Lennox-Gastaut syndrome | / | / |  |
| 11 | p.R1962C | 2m | Focal seizure | / | / | / |  |
| 12 | p.K3241T | 2y5m | Focal seizure | / | / | / |  |
| 13 | p.K3241T | 1y2m | Focal seizure | / | / | / |  |
| 14 | p.K3241T | 10y | Focal seizure | / | / | / |  |
| 15 | p.R3344Q | 5m | Focal seizure | / | / | / |  |
| 16 | p.K305N | 1y | / | / | / | controlled | Di Donato, 2018 |
| 17 | p.R309H | 5 m | / | / | / | 3 drugs therapy |  |
| 18 | p.R309H | 3 m | / | / | / | uncontrolled |  |
| 19 | p.R309HR | 3 m | / | / | / | controlled |  |
| 20 | p.V668D | 1y7 m | / | / | / | uncontrolled |  |
| 21 | p.R1623Q | 4 m | / | / | / | controlled |  |
| 22 | p.L2605del | 9 m | / | / | / | controlled |  |
| 23 | p.G3630S | 1 y | / | / | / | controlled |  |
| 24 | p.E3771K | 8 m | / | / | / | partially controlled |  |
| 25 | p.K3318N | 4 w | / | / | / | Uncontrolled |  |
| 26 | p.R3344W | 3 m | / | / | / | uncontrolled |  |
| 27 | p.R3344Q | 7 m | / | / | / | controlled |  |
| 28 | c.11941+2T>A | 6y 6mo | myoclonic jerks | / | / | Poorly controlled, |  |
| 29 | p.S1122P | / | / | epilepsy | / | / | Theunissen, et al,2018 |
| 30 | p.G3658E | / | / | epilepsy, | multifocal epileptiform discharges | / | Amabile, 2020 |
| 31 | p.E1564V | 2 m | myoclonic seizures；tonic seizures；focal impaired awareness seizures；atonic seizures | / | Interictal EEG showed waxing and waning of a waves in the frontal, temporal, and occipital areas, and high-amplitude rhythmic waves were frequently observed | CBZ, ZNS, CZP, VPA, LEV, PB  Not controlled | Matsumoto,2021 |
| 32 | p.L4179S | 7 y | generalized tonic–clonic seizures, myoclonic seizures，focal impaired awareness seizures | / | generalized spike-and-wave complexes and irregular poly-spikes and slow waves, predominantly in the left frontal area | VPA, lamotrigine, CLB, and LEV; Not controlled |  |
| 33 | p.R2244W | 13m | Epileptic spasms, focal seizure | / | / | / | Epi4K Consortium,2021 |
| 34 | p.G3658E | 6m | GTCS | / | an attenuated background of mixed theta and delta frequencies and semiperiodic spike and slow wave activity in the right temporal occipital region | Vigabatrin | Hertecant, 2017 |
| 35 | p.R1962C | 3m | Focal tonic/opisthotonic posturing. IS (6 mo) to multiple types | / | / | / | Elizabeth,2018 |
| 36 | p.F1093S | / | infantile spasm, EE | / | / | / | Helbig, 2016 |
| 37 | p.R3384Q | / | / | / | / | / | Chen, 2017 |
| 38 | p.D1062G | 17y | focal seizure; complex partial and secondary generalized seizures | / | / | treatment-refractory | Strickland, 2015 |
| 39 | p.M3392V | / | / | West syndrome | / | / | Lin, 2017 |
| 40 | p.V1116A | / | / | seizure | Generalized slowing | / | Amabile, 2020 |
| 41 | p.E1518K | 3y | generalized epileptic seizures | / | / | / | Willemsen, 2012 |
| 42 | p.R4449P | / | Focal seizure | / | / | / | Friedman, 2018 |
| 43 | p.R1603T | / | / | epilepsy | / | / | Soto, 2015 |

m : month; y : year; / : not mentioned
